# Fibrillary glomerulonephritis: A retrospective analysis of a case series from a tertiary center 

**DOI:** 10.5414/CNP104S10

**Published:** 2025-11-28

**Authors:** Ana Dovč, Željka Večerić-Haler, Nika Kojc, Špela Borštnar, Andrej Škoberne, Andreja Aleš Rigler

**Affiliations:** 1 Department of Nephrology, Division of Internal Medicine, University Medical Center Ljubljana,; 2 Faculty of Medicine, and; 3 Institute of Pathology, Faculty of Medicine, University of Ljubljana, Ljubljana, Slovenia

**Keywords:** fibrillary glomerulonephritis, rituximab, end-stage kidney disease, immune-complex glomerulonephritis, DNAJB9 staining

## Abstract

Introduction: Fibrillary glomerulonephritis is a rare diagnosis, and guidance on diagnosis and management is scant. We present a case series of patients with fibrillary glomerulonephritis from University Medical Center Ljubljana. Materials and methods: We conducted a retrospective analysis of patients with the diagnosis of fibrillary glomerulonephritis since 2006. We analyzed data on clinical presentation, treatment modalities, and kidney survival. For a subset of patients, data on DNAJB9 staining and markers of complement activation were also available. Results: We included 17 patients with fibrillary glomerulonephritis, 59% female, with a median age of 61 years (range 33 – 71). The most common clinical presentation was asymptomatic proteinuria with preserved kidney function (41%). Eight patients had complement activity testing performed, which revealed elevated serum C5b-9 at least at 1 time point in 5 patients (63%). One patient had positive anti-factor H antibodies, and 2 patients had positive anti-C1q antibodies. Nine patients had available staining results for DNAJB9, which was positive in all 9 cases. The median time of follow-up was 12 months. The most common form of treatment was with corticosteroids in 8 patients, followed by rituximab in 5 patients. Seven patients (41%) had at least partial remission with stabilization of kidney function, 8 patients (47%) reached end-stage kidney disease during follow-up. Due to the small number of patients, analysis of impact of treatment on kidney survival was not performed. Conclusion: Fibrillary glomerulonephritis is a heterogenous disease, and a significant number of patients present with a slowly progressive disease, warranting long-term treatment. New treatment strategies are necessary.

## Introduction 

Fibrillary glomerulonephritis (FGN) is a rare glomerular disease, representing ~ 0.5 – 1% of native kidney biopsies [1, 2, 3]. It is most commonly found in middle-aged adults, with a slight female predominance. Proteinuria is almost a universal finding, and a significant proportion (25 – 52%) of patients will present with nephrotic syndrome [3, 4, 5]. It is associated with systemic conditions, including diabetes mellitus, hepatitis C virus infection, autoimmune diseases, and malignancies in a minority of cases [6]. Coexistent glomerulopathies occur in a minority of cases; concurrent IgA nephropathy, although rare (~ 1.8%), is the second most frequent after diabetic nephropathy [7]. 

Histopathologically, FGN is an immune-complex glomerulonephritis (GN), characterized by randomly arranged nonbranching fibrils 10 – 30 nm in diameter, deposited in the mesangium and glomerular basement membrane as seen on electron microscopy. These fibrils are typically Congo red negative and positive for DnaJ homolog subfamily B member 9 (DNAJB9) staining, which may help with diagnosis in equivocal cases [4, 8, 9]. Immunofluorescence most often reveals smudgy mesangial and capillary wall staining for polyclonal IgG – predominantly IgG4 and/or IgG1 – with both κ and λ light chains. IgG and C3 staining is almost universally positive, while C1q staining is positive in a minority of cases, reflecting possible classic complement activation by IgG1 and IgG4 [8]. 

Clinically, the most common histologic pattern is mesangial proliferative GN, which is associated with better-preserved kidney function at diagnosis. Membranoproliferative GN (MPGN) morphology, which may represent an advanced stage, correlates with poorer outcomes. Crescents are present in up to 50% of biopsies, usually focal, and may be associated with a clinical presentation of rapidly progressive GN [1, 10, 11]. Prognosis is heterogeneous: while up to half of patients progress to end-stage kidney disease (ESKD) within several years, others exhibit a more indolent course. Risk factors for progression include reduced estimated glomerular filtration rate (eGFR) at presentation, male sex, older age, higher proteinuria, crescents, and glomerulosclerosis [3, 12]. 

There is currently no standard therapy for FGN [13]. Supportive management with renin–angiotensin–aldosterone system blockade and other anti-proteinuric measures is recommended for all patients. Immunosuppressive approaches such as rituximab, corticosteroids, cyclophosphamide, and mycophenolate mofetil have been used with variable results; a small prospective trial of rituximab showed proteinuria reduction without significant improvement in renal function [14], while a repository corticotropin injection with or without tacrolimus yielded at least a partial remission in 50% of patients at 1 year [15]. 

Here, we present a case series of 17 patients diagnosed with FGN at University Medical Center Ljubljana. 

## Materials and methods 

We reviewed the medical records of all adult patients with a histological diagnosis of FGN on native kidney biopsies performed since 2006. All biopsies were processed according to standard procedures, with light microscopy, immunofluorescence, and electron microscopy performed. For DNAJB9 immunohistochemistry, slides were stained with an anti-DNAJB9 rabbit polyclonal antibody (DNAJB9, Sigma-Aldrich, Darmstadt, Germany). FGN was defined by glomerular deposition of randomly oriented fibrils with a diameter of 10 – 30 nm that lack a hollow center. At the time of or following the diagnosis, all patients were screened for infection with the hepatitis B and C viruses and human immunodeficiency virus (HIV), autoimmune disorders, and malignancy. Baseline demographic data were collected, as well as laboratory data assessing kidney function at the time of biopsy and at follow-up clinics. A subset of patients had available data on complement activation testing (the degree of activation of classical, alternative, and lectin pathway, C5b-9 urine and serum levels, and the presence of anti-factor H, anti-C3nef, and anti-C1q antibodies). Clinical presentation was categorized in four different groups: asymptomatic proteinuria (with preserved kidney function), proteinuria with chronic kidney disease (CKD), nephrotic syndrome, and rapidly progressive GN. Clinical data concerning treatment and outcomes were collected up until the last available follow-up visit, reviewed on August 15, 2025. Time to last follow-up was defined as the time (in months) to last documented follow-up visit or the time to ESKD. 

Outcomes of interest included (in line with previously published data):

- Remission: reduction in proteinuria of ≥ 50% from the value at kidney biopsy and stable kidney function (< 25% decrease in eGFR from baseline).
- Persistent kidney dysfunction: no response according to previous criteria or progression of kidney disease but not ESKD at the last follow-up visit.
- ESKD or death.

Our study is in compliance with the Declaration of Helsinki and good clinical practice. The National Medical Ethics Committee of the Republic of Slovenia approved the conduct of this retrospective analysis (No. 0120-21/2018/5). 

## Results 

We retrospectively analyzed data of 17 patients with the diagnosis of FGN, with a median age of 61 years (range 33 – 71 years), of whom 59% were female. The most common comorbidity at presentation was arterial hypertension (41%), followed by diabetes (17%). Among associated conditions, 2 patients had a pre-existing autoimmune condition (1 patient had rheumatoid arthritis and 1 patient had both rheumatoid arthritis and multiple sclerosis). Three patients had a pre-existing malignancy (1 had a successfully treated papillary carcinoma of the bladder, 2 had chronic lymphocytic leukemia without specific treatment). One additional patient was discovered to have chronic lymphocytic leukemia at presentation. There were no patients with an associated infection with the hepatitis B or C virus, or HIV (Table 1). 

The most common clinical presentation was asymptomatic proteinuria with preserved kidney function (41%), followed by proteinuria in the setting of CKD (35%). Only 3 patients (17%) presented with nephrotic syndrome. One patient presented as rapidly progressive GN and was discovered to have concurrent GN associated with anti-neutrophil cytoplasmic antibodies (ANCA). Four additional patients had positive immunoserology (3 patients had positive antinuclear antibodies, 2 patients had positive anticardiolipin antibodies, 1 patient had low positive cryoglobulins polyclonal IgG) – after careful review of the clinical data, none of these patients were determined to have systemic connective tissue disease. Two patients had monoclonal gammopathy of undetermined significance, both of whom had polyclonal IgG deposition on kidney biopsy. Eight patients had complement activity testing performed, which revealed elevated serum C5b-9 at least at 1 time point in 5 patients (63%). One patient had positive anti-factor H antibodies, and 2 patients had positive anti-C1q antibodies. Three patients had concurrent acute interstitial nephritis found on kidney biopsy. Nine patients had available staining results for DNAJB9, which was positive in all 9 cases. 

The median time of follow-up was 12 months, with the average follow-up time being 39 months. Ten patients (59%) received specific treatment with corticosteroids, mycophenolate mofetil, cyclophosphamide or rituximab, alone or in combinations. The most common form of treatment was with corticosteroids (either oral or pulse) in 8 patients (80%), followed by rituximab in 5 patients (50%). Two patients received chemotherapy for either suspected monoclonal gammopathy of renal significance or chronic lymphocytic leukemia. 

Seven patients (41%) had at least partial remission with stabilization of kidney function. Two patients had slowly progressive CKD (including patient with concurrent ANCA-associated GN). Six patients (35%) had to start kidney replacement therapy (KRT) within 1 year of diagnosis due to advanced kidney disease, of which two required KRT at presentation and 3 reached ESKD within 2 months of diagnosis. Two patients reached ESKD at 10 and 13 years after diagnosis (both presented with asymptomatic proteinuria at initial biopsy). One patient had a kidney transplant during the time of observation, with excellent graft function. An indication biopsy 10 years after transplant due to rising proteinuria revealed a recurrence of the disease. 

Due to a small number of patients, analysis of impact of treatment on kidney survival was not performed. 

## Discussion 

We retrospectively analysed the treatment and outcomes of 17 adult patients with fibrillary GN diagnosed at our center since 2006. Eight patients (47%) reached ESKD within the follow-up period with a mean time of 37 months, which is in line with previous findings [1, 3, 7, 16]. However, patients with FGN seem to have a remarkably heterogeneous clinical presentation, and determining the average time to ESKD poorly reflects their prognosis. In our cohort, a significant number of patients presented with advanced CKD or required KRT at the time of biopsy, skewing results towards worse outcomes. 

Among patients who presented with proteinuria or nephrotic syndrome (either with preserved or mildly impaired kidney function), the disease course was slowly progressive. Only 2 of 9 such patients progressed to ESKD during the time of observation: 1 after 10 years due to serum sickness following rituximab treatment, the other after 13 years. Unfortunately, the latter was lost to follow-up after a few years, and presented with advanced kidney dysfunction 13 years after the initial diagnosis, requiring immediate start of KRT. A kidney biopsy performed at the time revealed MPGN pattern of injury (in contrast to mesangial proliferation seen on initial diagnosis), which might lend some credence to the hypothesis that both patterns of injury are part of a spectrum of FGN, where MPGN pattern might be associated with a diagnosis later in the disease course [3, 10]. 

An interesting observation from our cohort is that in recent years, patients receive a diagnosis earlier in the disease course while kidney function is still preserved. Such cases pose a curious dilemma: it seems inappropriate to employ immunosuppressive treatment with significant risk of adverse effects for a patient with non-nephrotic proteinuria and a slowly progressive course. When patients in our cohort have received specific treatment (mainly with rituximab), remission occurred slowly and was in most cases only partial. This is an expected finding because structured deposits such as in fibrillary GN are slow to clear, and in the absence of a specific biomarker or performing a repeat biopsy, response to treatment is difficult to determine. 

There is a paucity of evidence-based treatment options for fibrillary GN, but the issue may also lie in the diversity of the clinical presentation that argues against a one-size-fits-all approach. FGN is, in its essence, an immune-complex GN. The discovery of DNAJB9 positivity might be a clue to its pathophysiology. However, it is likely that FGN – which is a histopathologic diagnosis – comprises of different etiologies with a unifying feature of DNAJB9 deposition and fibril formation. In recent years, we have new diagnostic possibilities of testing for complement activity, which enabled us to discover signs of complement dysregulation in a number of patients with FGN; namely elevated levels of C5b9 in serum in 5 patients and autoantibodies against factor H in 1 patient. Two patients were also positive for anti-C1q antibodies. None of these 3 patients had systemic disease, known to be associated with FGN. Thus, in such cases, we might regard this disease as a fibrillary form of idiopathic immune-complex GN, which raises the possibility that anticomplement therapy might be an effective form of treatment [17]. 

Lastly, our study is limited by its retrospective nature and small number of patients. Because FGN is rare, randomized clinical trials of new treatments are scarce (at the time of writing, only one clinical trial in fibrillary GN was registered at clinicaltrials.gov as recruiting). Perhaps a more granular analysis of already published case reports and case series might reveal new insights on which patients might benefit the most from different specific treatments. 

## Conclusion 

In recent years, an expanding awareness of kidney disease has enabled clinicians to refer patients for kidney biopsy sooner, facilitating an earlier diagnosis of FGN. At least in our case series, patients with the diagnosis established when kidney function is still preserved had a slowly progressive disease, which differs from the previous notion of FGN as having an almost invariably poor prognosis. In parallel to diseases such as lupus nephritis and IgA nephropathy, expanding knowledge of pathophysiology may reveal new treatment possibilities, and a repeat biopsy should be considered to evaluate treatment response better. Further research is needed to clarify the mechanistic role of DNAJB9, establish optimal treatment strategies, and determine the long-term outcomes of affected patients. 

## Authors’ contributions 

Conceptualization, AD, ŠB, ŽVH, NK, AŠ, and AAR; acquisition of patient data, ŠB, ŽVH, AŠ, NK and AD; formal analysis, ŠB and AD; writing – original draft preparation, AD; writing – review and editing, AD, ŠB, ŽVH, NK, AŠ, and AAR. All authors read and approved the final version of the manuscript. 

## Funding 

This study received no funding. 

## Conflict of interest 

The authors declare no conflict of interest. 

**Table 1.** Demographic data and clinical characteristics of patients diagnosed with fibrillary glomerulonephritis at our center from 2006.

	Year of diagnosis	Age at diagnosis	Sex	Comorbidities	Serum creatinine at initial biopsy (μmol/L)	Proteinuria at initial biopsy (g/day)	Red blood cells in urine sediment at initial biopsy (/400x)	Histopathology on initial biopsy	Specific treatment	Time of follow-up/time to ESKD (months)	Outcome/serum creatinine at last follow-up (μmol/L)	Special remarks
1	2006	58	F	Rheumatoid arthritis, multiple sclerosis, bladder carcinoma	60	Unavailable	Unavailable	Mesangial proliferation, crescents	CS, MMF	159	ESKD	Membranoproliferative pattern on repeat biopsy
2	2010	55	F	Chronic lymphocytic leukemia	63	3.0	Unavailable	Mesangial proliferation, crescents	RTX	122	ESKD	Serum sickness following RTX treatment
3	2011	44	F	None	Unavailable	Unavailable	Unavailable	Mesangial and endocapillary proliferation, crescents (35%)	Supportive	1	ESKD	Recurrent fibrillary GN 10 years after transplantation
4	2012	60	M	HTN, DM	667	6.4	25	Mesangial proliferation, crescents (60%)	CS	0	ESKD	Concurrent acute tubulointerstitial nephritis
5	2014	71	F	HTN, chronic lymphocytic leukemia	221	4.4	13	Membranoproliferative, crescents (6%), monotypic deposits IgG/λ	R-CHOP	1	ESKD	Concurrent acute tubulointerstitial nephritis
6	2015	35	M	Horseshoe kidney	97	2.9	8	Immune-complex on immunofluorescence, no proliferation	RTX	122	126	Slowly progressive chronic kidney disease
7	2016	61	F	Monoclonal gammopathy of uknown significance	1.186	13.5	24	Crescents (100%)	CS, bortezomib	0	ESKD	Treated as monoclonal gammopathy of renal significance
8	2018	66	F	HTN, DM	94	1.4	1	Mesangial proliferation	Supportive	87	106	Concurrent acute tubulointerstitial nephritis
9	2018	62	M	HTN, liver cirrhosis	703	4.7	26	Mesangial and endocapiilary proiliferation, crescents (18%)	Supportive	2	ESKD	None
10	2018	63	M	Spinal stenosis, low-grade cryoglobulinemia	158	4.13	8	Mesangial proliferation, full-house deposits on immunofluorescence, crescents (8%)	CS, RTX	82	375	Anti-C1q antibodies, anti-cardiolipin antibodies
11	2019	64	M	Rheumatoid arthritis, HTN, monoclonal gammopathy of uknown significance	284	2.8	9	Mesangial and endocapillary proliferation, one crescent (2%)	Supportive	9	ESKD	None
12	2019	62	M	HTN, DM	391	8.1	36	Immune-complex GN, crescents (24%)	CS, cyclophosphamide	75	496	Concurrent MPO-ANCA-associated GN with several relapses
13	2023	64	F	Asthma, chronic lymphocytic leukemia	71	6.6	70	Mesangial proliferation, monotypic deposits IgG1/λ	CS, RTX	26	61	Full remission
14	2024	70	F	Chronic obstructive pulmonary disease	88	0.6	0	Mesangial proliferation	Supportive	15	84	Anti-C1q antibodies
15	2024	33	F	None	63	1.1	7	Mesangial proliferation	Supportive	7	64	Anti-factor H antibodies
16	2024	56	F	HTN	93	2.3	14	Mesangial proliferation	Supportive	9	86	Partial remission
17	2025	47	M	HTN, hypothyroidism	128	6.9	8	Mesangial proliferation	CS, MMF	2	187	Partial remission of nephrotic syndrome

CS = corticosteroids; DM = diabetes mellitus; ESKD = end-stage kidney disease; F = female; HTN = arterial hypertension; GN = glomerulonephritis; M = male; MMF = mycophenolate mofetil; MPO-ANCA = anti-neutrophil cytoplasmic antibodies against myeloperoxidase; R-CHOP = rituximab, cyclophosphamide, doxorubicin, vincristine, corticosteroids; RTX = rituximab.
